# School-Based Family-Oriented Health Interventions to Promote Physical Activity in Children and Adolescents: A Systematic Review

**DOI:** 10.1177/08901171221113836

**Published:** 2022-11-22

**Authors:** Francisco Santos, Honorato Sousa, Élvio Rúbio Gouveia, Helder Lopes, Miguel Peralta, João Martins, Eugenia Murawska-Ciałowicz, Grzegorz Żurek, Adilson Marques

**Affiliations:** 1 Department of Physical Education and Sport, 56057 University de Madeira, Funchal, Portugal; 2 LARSYS, Interactive Technologies Institute, Funchal, Portugal; 3 Research Center in Sports Sciences, Health Sciences, and Human Development (CIDESD), Vila Real, Portugal; 4 CIPER, Faculdade de Motricidade Humana, Universidade de Lisboa, Lisboa, Portugal; 5 ISAMB, 70882 University of Lisbon, Lisbon, Portugal; 6 Physiology and Biochemistry Department, 49938 University School of Physical Education in Wrocław, Wrocław, Poland; 7 Department of Biostructure, 49938 University School of Physical Education, Wroclaw, Poland

**Keywords:** physical activity program, school context, family participation, adolescents

## Abstract

**Objective:**

This study aimed to systematically review and analyse intervention programs in a school context centred on the family, focused on increasing youths' physical activity.

**Data source:**

The research was carried out in the PubMed, Scopus and Web of Science databases.

**Study inclusion criteria:**

Studies were included if participants were children or adolescents, focusing on school-based intervention studies with parental involvement and physical activity, sedentary behaviour or physical fitness outcomes.

**Data extraction:**

The search was performed according to the PRISMA protocol. A total of 416 articles were identified. After being considered for eligibility and duplicates, 22 studies were identified as relevant for inclusion.

**Data synthesis:**

Sample and intervention characteristics, objective, the role of the family, outcomes measures, main findings regarding the outcomes and risk of bias.

**Results:**

Ten studies reported improvements in physical activity, 6 in sedentary behaviour and 9 in the components of physical fitness and/or skills related to healthy behaviours and lifestyles. Most of the interventions adopted a multidisciplinary and multi-component approach.

**Conclusions:**

Most interventions employed a school’s multidisciplinary/multi-component approach to promoting physical activity, nutrition, and general education for healthier lifestyle behaviours. The impact of school-based interventions involving families on youth’s physical activity levels is still a relatively emerging theme. Further research is needed given the diversity of the intervention’s characteristics and the disparity in the results’ efficacy.

## Objective

Implementing health behaviours, such as physical activity, should be established as soon as possible in the life cycle. Thus, promoting physical activity should be consistent throughout childhood and adolescence. Due to the significant amount of time children and adolescents spend at school, this setting has been considered a key opportunity for nearly all school-aged children and adolescents to access health-enhancing physical activity.^1,2^ Although increasing evidence suggests that schools can play an important role in promoting active lifestyles, there is also evidence stating that the effects are generally short-lived.^3-6^ In fact, it is still unclear which factors influence the effectiveness of interventions for promoting medium- and long-term healthy behaviours, such as physical activity engagement.^7,8^ Thus, there is still a need to understand better what aspects of the social and built environment are associated with physical activity participation since these are critical but less explored factors to consider in designing successful physical activity programs in the school setting.

Family involvement may play a fundamental role in adopting and maintaining active lifestyles that cannot be ignored in the school setting when considering children and adolescents.^9,10^ Multidisciplinary interventions in the school setting involving the school community and students’ families have the potential to promote physical activity and healthy lifestyles among children, adolescents, and their relatives.^11,12^ Family support, encouragement, and the availability to monitor children’s physical activity are critical factors that influence children and adolescents attitude towards physical activity.^13-15^ Furthermore, some evidence suggests that physical activity interventions integrating the family lead to longer-lasting effects. Even within interventions incorporating the family, those where parents participate directly and regularly in the activities show better results when compared to interventions where parents participate indirectly.^8,16-18^

Despite the literature suggesting the beneficial effects of family involvement in school-based physical activity interventions, there is still no systematized evidence on how family interventions influence children and adolescents' physical activity levels. Thus, the objective of this study was to systematically review the effects of school-based interventions involving the family in promoting physical activity and/or reducing sedentary behaviour among children and adolescents to understand its effects better. Also, this systematic review will allow physical education and general education professionals to identify important factors to consider in developing and implementing physical activity interventions involving the family in the school context.

## Methods

This systematic review followed the Preferred Reporting Items for Systematic Reviews and Meta-Analyses (PRISMA) 2020 guidelines.^19^

### Search Strategy

The electronic databases considered in this systematic review were PubMed, Scopus, and Web of Science were searched for relevant records on the 30th of April 2021. The following terms were searched in the title: “physical activity” OR sport* OR exercise OR fitness OR sedentary OR “motor skill* AND school AND intervention* OR program* OR protocol* OR RCT OR “randomized controlled trial” OR experimental AND health*.

### Study Selection

Primary source articles published in peer-reviewed scientific journals were considered eligible until the 30^th^ of April 2021. Articles were included in the systematic review if they met the following criteria according to PICOS (participants, intervention, comparison, outcome, study design) guidelines: (1) participants were children, adolescents, and their relatives; (2) school-based intervention studies with parental involvement; (3) any comparison; (4) physical activity, sedentary behaviour, or physical fitness outcomes; and (5) RCT design studies. Duplicated records from the database search were removed. Afterward, the title and abstract were independently screened by 2 authors (FS, HS) for eligibility. A complete reading of the eligible records was carried out to consider inclusion in the systematic review. The same 2 authors reviewed potential studies, and the inclusion and exclusion decisions were made by consensus.

### Data Extraction and Harmonization

Two authors (FS, HS) performed data extraction and harmonization using a standardized approach with a consensus. Relevant information extracted included: sample characteristics (number, age, and parents where the study was carried out), study features (duration, main characteristics of the intervention, and how the parents were involved), measures/instruments used (only those that mediate physical activity and/or sedentary behaviour were considered) and the main results.

### Study Quality and Risk of Bias

#### The Effective Public Health Practice Project (EPHPP) Quality Assessment Tool for

Quantitative studies were used to assess study quality.^20^ This instrument has 6 components that consider selection bias, including study design, confounding factors, data collection methods/instruments, whether raters and participants were “blinded”, reports of withdrawals, and dropouts. According to the specific criteria, scores of weak, moderate, or strong were assigned to each category. Study quality was independently assessed by 2 authors (FS, HS). The discrepancies were discussed and decided by agreement.

## Results

### Study Selection

**Figure 1.**
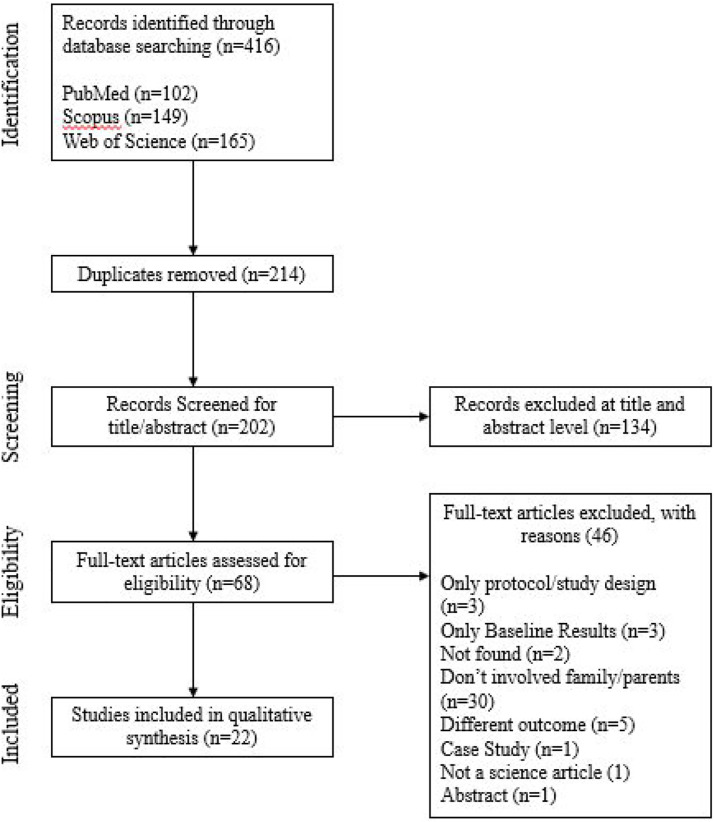
Flowchart of the study selection.

The flowchart of the study selection process is shown in Figure 1. Through the search carried out in the databases, 416 articles were identified. Of these, 202 were considered for eligibility after duplicates were removed (n=214). A total of 134 studies were eliminated in the title and abstract screening phase. Lastly, the full text of 68 studies was fully assessed, and 22 were elected as relevant for inclusion.

### Study Quality and Risk of Bias

The summary of the study quality assessment is presented in Table 1. Of the 22 studies included in the review, one was classified as having strong methodological quality,^21^ eleven with moderate methodological quality,^22-32^ and ten with poor quality.^33-42^ In terms of parameters evaluated, in the selection of bias, only 4 studies were evaluated as good,^21,25,28,32^ because they are considered representative of the target population with a participation rate of over 80%. Study designs were classified as strong when RCTS or Controlled Clinical Trials (n=12)^21-26,28,29,32,33,38,39^ and fair when they were Cohort analytic, case-control, cohort, or an interrupted time series (n=10).^27,30,31,34-37,40-42^ Regarding confounders parameter, 9 studies reported no baseline differences between groups^22,23,25,26,30-32,37,39^ and 8 studies accounted for at least 80% with significant confounders.^21,24,27-29,33,36,38^ Cohorts and other types of interventions performed with only 1 group were not considered.^34,35,40-42^ Concerning the blinding part, studies were assessed as good when they blinded assessor and participants,^21^ and fair when they blinded 1 of the 2 criteria.^30,38^ The data collection methods demonstrated that almost studies (n=21) offered evidence of the validity and reliability of the reported outcome measures. Lastly, in terms of withdrawals and dropouts, studies were classified as good when the follow-up rate was higher than 80%,^23,25,27,28,30,31,42^ fair when the follow-up rate was between 60-79%,^21,22,24,26,29,32,41^ and poor when the follow-up rate was <60% or withdrawals and dropouts were not described.^33-40^

**Table 1.** Studies Methodological Quality Assessment.

Authors	Selection Bias			Design					Confounders			Blinding			Data Collection Methods			Withdrawals and drop-Outs			Final
	Q1	Q2	R	Q1	Q2	Q3	Q4	R	Q1	Q2	R	Q1	Q2	R	Q1	Q2	R	Q1	Q2	R	
Kobel et al, 2020	1	3	F	1	Yes	Yes	Yes	G	2	n/a	G	3	3	P	1	1	G	3	2	F	Moderate
Wieland et al, 2020	3	5	P	5	No	n/a	n/a	F	n/a	n/a	n/a	3	3	P	1	1	G	3	4	P	Weak
Bartelink et al, 2019	2	2	F	3	No	n/a	n/a	F	1	1	G	3	3	P	1	1	G	1	1	G	Moderate
Pearce and Dollman, 2019	3	5	P	3	No	n/a	n/a	F	1	1	G	3	3	P	1	1	G	1	3	P	Weak
Robbins et al, 2018	2	3	P	2	Yes	Yes	Yes	G	1	1	G	2	3	F	1	1	G	1	3	P	Weak
Wang et al, 2018	1	1	G	1	Yes	Yes	Yes	G	2	n/a	G	3	3	P	1	1	G	1	1	G	Moderate
Cochrane and Davey, 2017	1	3	F	5	No	n/a	n/a	F	n/a	n/a	n/a	3	3	P	3	3	P	1	2	F	Weak
Rabiei et al, 2017	3	5	P	3	No	n/a	n/a	F	2	n/a	G	3	3	P	1	1	G	2	4	P	Weak
Andrade et al, 2016	1	1	G	1	Yes	Yes	Yes	G	1	1	G	2	2	G	1	1	G	1	2	F	Strong
Uys et al, 2016	1	3	F	2	Yes	Yes	Yes	G	1	1	G	3	3	P	1	1	G	1	2	F	Moderate
Nyberg et al, 2015	2	2	F	1	Yes	Yes	Yes	G	2	n/a	G	3	3	P	1	1	G	1	1	G	Moderate
Robles et al, 2014	3	1	P	5	No	n/a	n/a	F	n/a	n/a	n/a	3	3	P	1	1	G	1	1	G	Weak
Casey et al, 2014	2	5	F	1	Yes	Yes	Yes	G	1	1	G	3	3	P	1	1	G	2	3	P	Weak
Habib Mourad et al, 2014	1	1	G	2	Yes	Yes	Yes	G	1	1	G	3	3	P	1	1	G	1	1	G	Moderate
Fairclough et al, 2013	1	2	F	1	Yes	Yes	Yes	G	1	1	G	1	3	P	1	1	G	1	2	F	Moderate
Muros et al, 2013	3	2	P	3	No	n/a	n/a	F	2	n/a	G	2	3	F	1	1	G	1	1	G	Moderate
Wright et al, 2013	2	1	F	1	Yes	Yes	No	G	2	n/a	G	3	3	P	1	1	G	1	4	P	Weak
Coen et al, 2012	1	3	F	1	Yes	Yes	No	G	2	n/a	G	3	3	P	1	1	G	1	2	F	Moderate
Kargarfard et al, 2012	1	5	F	3	No	n/a	n/a	F	2	n/a	G	3	3	P	1	1	G	1	1	G	Moderate
Dzewaltowski et al, 2009	1	1	G	1	Yes	Yes	Yes	G	2	1	G	3	3	P	1	1	G	1	2	F	Moderate
Jan et al, 2009	1	5	F	5	No	n/a	n/a	F	n/a	n/a	n/a	3	3	P	1	1	G	1	4	P	Weak
Engels et al, 2005	3	5	P	5	No	n/a	n/a	F	n/a	n/a	n/a	3	3	P	1	1	G	3	4	P	Weak

Abbreviation: R, rating of components; F, fair; G, good; P, poor/weak

### Intervention Characteristics

The characteristics of each study intervention are presented in Table 2. Considering all studies on this systematic review, 28 760 students were included. The smallest sample was found in the Robles et al study (n=33), and the biggest sample was seen in the Wang et al study (n=9858). Interventions covered different ages and school years. Thus, 7 studies were carried out in primary schools^22,23,26,27,29,30,41^ and 9 in elementary or high schools.^21,28,31-34,37,38,40^ There were 8 studies in which the intervention population encompasses 1 or more education levels.^24,25,35,36,39,42^ The interventions duration varied widely: <3 months, 10 studies^28,30,31,34-39,42^; 3-11 months, three studies^23,29,40^; and ≥12 months, nine studies.^21,22,24-27,32,33,41^

**Table 2.** Characteristics and Main Results of the Studies Included in The Systematic Review.

Autor	Objective	Sample characteristics (N; age; country)	Intervention (duration; name and Main Characteristics of the Program; parents involvement)	Outcome (measures)	Main Results	Effects in Terms of the Main Outcomes
Kobel et al, 2020	To investigate the effects “Join the healthy Boat” program on children’s sedentary behaviour	IG 102 (46.1% male); CG 52 (67% male); 5-8 years; South-West Germany	1 school year duration. “Join the healthy Boat” program included train teachers to change the school environment as well as their teaching to promote PA, a healthy diet, and active leisure time. The program includes 20 lessons for 1 year focused on PA and health education and 2 exercises every day (5-10 min). Parents participated in the “family homework”, in which the children exercise together with their parents at home	Sedentary time (multi-sensor device measuring HR, METs and Sleeping time). Screen time (parental questionnaire)	No significant improvements were seen in children’s sedentary time. Sedentary time was higher at weekends. Parent-reported screen time decreased in the intervention group	PA: Nothing, SB:Positive
						F/S: Nothing
Wieland et al, 2020	To develop and implement a multi-component PA and healthy eating intervention	61 children (mean age of 10.4 years), Rochester, Minnesota, United States	6 months duration. The multi-component PA and healthy eating program is an afterschool program that included eating and PA policy implementation, staff training, a challenge, and self-monitoring program for healthy behaviors, a peer-coaching program for healthy behaviors, and a social marketing campaign. Parents were involved in home self-monitoring	Self-efficacy, motivation, and social support for PA (Kinetic activity monitor accelerometer)	The results showed statistically significant improvements in self-efficacy and motivation for PA. Screen time improved but was not statistically different from baseline. No improvements were found in perceived social support and objectively measured PA.	PA: Nothing, SB:Nothing, F/S: Positive
	Afterschool program					
Bartelink et al, 2019	To examine the effects of HPSF on children’s dietary and PA behaviours after 1 and 2 years’ follow-up	1676 (47.4% boys); IG full 537; IG Partial 478; CG 661; Mean age 7.5; Parkstad, Netherlands	2 years intervention. “The healthy primary school of the future (HPSF) program” included changes in all aspects of the school system, such as a free healthy lunch each day and structured PA sessions after lunch (1 or 2 times/week). The collaboration of sports clubs and other external partners to offer specific activities was included. Children’s focus group, to hear the opinion of the children regarding the program. Parents filled a digital questionnaire of demographic information, household income, and children’s health behaviours	SB, LIPA, MVPA (accelerometer used 7 days)	Significantly favourable intervention effects were found in the accelerometery data in the full HPSF vs control schools. The percentage time spent sedentary had decreased more and the percentage time spent in light PA had increased more in children of the full HPSF compared with control schools	PA: Positive
						SB: Positive
						F/S: Nothing
Pearce and Dollman, 2019	To develop and evaluate a multicomponent school and home-based PA intervention. And determine the psychological variables that influence PA.	IG 63; CG 84; (total of 147 students, 55% being girls); 8- 13 years; Northern suburbs of Adelaide, Australia	10-week intervention and other 10 weeks to follow up. This multicomponent school and Home-based PA were based on 10x one-hour school-based training sessions consisting of 15-20 min of classroom training, and 40-45 min of PA was delivered by a teacher and physiotherapist; ‘passport to fun’ home-based activity booklet to encourage children’s daily participation in PA with their parents, guardians, siblings, and friends. 4x one-hour parent workshops, to support the SAKG program and promote awareness of a healthy diet and the importance of exercise, were delivered by a trained nutritionist and educator. Parents participated in a focus group, to debate the program	Enjoyment of PA and Perceived barriers to PA (questionnaires). PA (accelerometers and questionnaire)	73% of the children with complete data sets at the intervention school did not increase MVPA after-school period (3 p.m. to 6 p.m.) or over the whole day or during school break time immediately following the intervention or at follow-up. Overall, 59% of boys attained more than double the recommended 120 min of MVPA each day compared to 42% of girls	PA: Positive
						SB: Positive
						F/S: Nothing
Robbins et al, 2018	To evaluate the feasibility and acceptability of a school and home-based intervention and explore any effect of the intervention on adolescents’ percent body fat and BMI.	IG 39 adolescents and 38 parents; CG 45 adolescents and 43 parents; 10-13 years; Midwestern, United States	12-weeks intervention. GOAL included a three-component intervention: (a) Parent–adolescent dyad meeting to assist parents in helping their adolescents increase PA and healthy eating	MVPA (accelerometer-measured). PA self-efficacy, social support for PA, motivation for PA (questionnaires)	Intervention adolescents had greater autonomous motivation for PA. Although between-group differences were not significant, close-to-moderate effect sizes resulted for accelerometer-measured MVPA.	SB: Nothing
			(b) After-school GOAL club for PA, healthy eating, and cooking skill development for adolescents [120-min GOAL Club twice a week for 9 weeks (total 18 events]; (3) Facebook participation—parents together with adolescents (information, motivational messages, and behavioural strategies to help them assist their adolescents with increasing PA and healthy eating)			F/S: Positive
Wang et al, 2018	To develop a community-based PA intervention program aiming at childhood obesity prevention	IG 5275 (53.2% boys); CG 4583 (52.8% boys); 10.5 ± .01 years old; Nanjing in China	1 year intervention. School-based YOG-Obesity intervention comprises a school-based intervention and family involved Enrichment. In the school-based intervention the following activities were integrated: (a) School environment support (ie, Posters/slogans; instruments, news Leaflets; (b) Classroom curriculum. The family involved Enrichment included (a) Fun events (ie, Housework week; Walk-to -school week; No TV week; Contest; live show); (b) parents’ Class; (c) Parent–child home assignments	PA questionnaire, CPAIQ.	Compared with the baseline, PA level increased by 33.13 min per week in the intervention group but decreased by 1.76 min per week in the control group; after adjustment for potential confounders, compared with the control group, the intervention group were more likely to have increased time of PA.	PA: Positive
						SB: Nothing
						F/S: Nothing
Cochrane and Davey, 2017	To investigate the effect of the HEELP” program to encourage the engagement in PA and to improve eating behaviours	709; 5-10 years old; Canberra, Australia	4 years intervention. The HEELP program is an after-school program to encourage children and their parents to become more engaged in PA, to educate them about nutrition and healthy life. This program included 10 hours (7×90 minutes in the first 2 years and 8×75 minutes in the last 2 years). Each session included physically active and lifestyle educational games. Home-based activities were also provided each week to encourage family engagement with healthier lifestyle choices	Physical fitness components: HEELP participants Survey (fulfilled by the parents, measured the perception that they have form their children’s PA and other things)	HEELP survey shows that the parents noticed that their children were more physically active (51%). 45% of children improved cardiorespiratory fitness, standing long jump, and grip strength. In the families where noted that they were more active as a family (41%) and a reduction in screen time (14%)	PA: Positive
						SB: Positive
						F/S: Nothing
Rabiei et al, 2017	To evaluate the effect of PA programs on self-esteem and BMI of overweight adolescent girls	IG (70 girls); CG (70 girls); second-grade public high school; Isfahan, Iran	3-months intervention. Six sessions each 60-minute were conducted with emphasis on the diet to control weight in overweight and at-risk adolescents. The educational program was held using direct education, speech, and active participation of study subject (question and answer), using educational slides, as well as indirect education, by providing educational pamphlets. Parents had 2 educational sessions 60- minute-long and fulfilled the questionnaire	Based health Belief Model, to measure weekly PA.	Perceived benefits, perceived obstacles, self-efficacy, scores of PA were significantly higher in the case group immediately after and 3 months the intervention compared with CG. In terms of performance status of students in physical activities, it was significantly higher in the case group 3 months after the intervention compared with CG. In terms of parents’ performance regarding PA of their adolescent, no significant difference immediately after the intervention and 3 months after the intervention was seen	PA: Positive, SB: Nothing, F/S: Nothing
Andrade et al, 2016	To assess the differential effect of ACTVITAL” program in overweight and low-fit adolescents in terms of physical fitness and PA.	1440 adolescents; 12-15 years old; Cuenca, Ecuador	28 months intervention. “ACTVITAL” program, is based in individual strategy included the delivery of educational package organized at classroom level to promote healthy diet and an active lifestyle. The environmental strategy included workshops for parents, social events, and environmental modification (providing a walking trail in each school). Parents participate in the workshops, and fellfield the questionnaire to measure satisfaction and to get general feedback of the workshops	Physical fitness (EUROFIT). Sedentary behaviour (questionnaire). PA (Accelerometers)	Low-fit and overweight adolescents respond differently to ACTIVITAL program. Adolescents with poor physical fitness showed a higher improvement of muscular strength (vertical jump) compared to fit adolescents, after the intervention program. Overweight adolescents had a significantly lower increase in the time needed for the speed shuttle run test compared to normal-weight and underweight adolescents. The proportion of students achieving over 60 min of moderate-to-vigorous PA/day was not significantly different according to weight or fitness status	PA: Nothing, SB: Nothing, F/S: Positive
Uys et al, 2016	To assess the impact of “HealthKick” program targeting healthy eating and PA on physical fitness levels, and PA-related knowledge, attitudes, and behaviour	Baseline (IG 503/CG 499); year 1 (IG 526/CG 546); year 2 (IG 532/CG 556); Mean age 9,93 years old; Western Cape, South Africa	3 years intervention. “HealthKick” program, developed for health promotion in low-income communities. Based on the social ecological Model (intrapersonal, interpersonal, organizational community), the intervention schools received a HealthKick toolkit which contained an educator’s manual, a curriculum manual, a resource box, and a PA resource bin. Does not specify how the parents were involved	Physical fitness components. The general attitude towards PA, PA knowledge, social support, self-efficacy, perceived barriers, and enjoyment (KAB questionnaire)	No overall improvement in physical fitness was found. The sit-ups score improved significantly in the intervention group (P < .05). No overall intervention effects were found on the determinants of PA behaviour. Knowledge improved in both the intervention (P = .005) and control groups (P < .001)	PA: Nothing, SB: Nothing, F/S: Positiv.e
Nyberg et al, 2015	To develop and evaluate the effectiveness of a parental support programme to promote healthy dietary and PA habits and to prevent overweight and obesity	IG 129 (68 boys and 61 girls); CG 112 (55 boys and 57 girls); 6 years old, Stockholm County, Sweden	6 months. The healthy school Start study included: 1) health information, 2) motivational Interviewing with parents and 3) Teacher-led classroom activities. Parental were involved by (1) Brochure information increase parental knowledge on how to promote children’s dietary and PA habits; (2) motivational interview, to increase parental care and control and self-efficacy to provide support for healthy eating and PA to the child; (3) participated in homework tasks with their children	PA (accelerometry). PA habits, SB and sleep time assessed by EPAQ.	There was no significant intervention effect in the primary outcome PA. Sub-group analyses showed a significant gender-group interaction in total PA, with girls in the intervention group demonstrating higher total PA during weekends, as well as in sedentary time. The boys showed more sedentary time in the intervention group	PA: Nothing, SB: Nothing, F/S: Nothing
Robles et al, 2014	To determine whether a health-care professional out-of school time PA and nutrition education community program with vigorous intensity PA could influence the Physiologic and anthropometric measurements and the after-school PA.	33 children, 8-11years old; Bailey Country, Texas	12 weeks intervention. The pharmacy healthcare professional out-of-school time vigorous PA and nutrition education program comprises 2x60min a week for 1 month, of 15 min of alternating PA and nutrition education with 45 min of VPA. At the end of each week, they participated in a track meet. The parents complete the demographic questionnaire and cheered on the participants during the family-attended track meets	After-school student questionnaire (knowledge of food modifications, PA, video game, computer, and television use). Physiologic and anthropometric parameters	Positive survey results at 3 months indicated a decrease in fried/sweet foods; increase in exercise; decreases in video games and computer use; and a change in knowledge regarding the selection of the most healthy food group servings per day	PA: Positive, SB: Positive, F/S: Positive
Casey et al, 2014	To evaluate the effectiveness of a school-community linked PA-promotion intervention program targeting adolescent girls living in low-SES, On their HRQoL, levels of PA, and potential mediators of PA.	IG 362 girls; CG 259 girls; mean age 13,4 years old; Australian rural and regional communities	12 months intervention. This school-community-linked PA-promotion program included a school PE component that incorporated student centred teaching approaches and behavioural skill development. The PE component involved students participating in 2 6-session units each designed as a session per week during their ‘normal’ PE class time, which ranged from 57 to 100 minutes. The 2 units were a sport unit (tennis or football) and a recreational unit (YMCA). The influence of family and friends on PA and sport participation was measured using a modified version of questions on family and peer influences	HRQoL; PA level (self-reported using the PDPAR-24, questionnaire)	For HRQoL, after adjustment for baseline scores, the intervention group showed significantly higher scores on all 3 PedsQL scores than the control group. The three-group analysis found intervention non-completers had significantly higher PedsQL scores than controls. There were no significant differences for any PA measure. Intervention completers had significantly higher scores than non-completers and controls for some mediator variables (eg self-efficacy, behavioural control).	PA: Nothing, SB: Nothing, F/S: Positive
Habib Mourad et al, 2014	To evaluate the feasibility and effectiveness of a multicomponent school-based intervention to promote healthy eating and PA (and prevent obesity) with school children	IG 193; CG 181; 9-11 years old; Beirut, Lebanon	3 months intervention. “Health-E-PALS” program, was based on the constructs of the social Cognitive Theory. The program included 12 culturally appropriate classroom sessions using fun and interactive activities once a week and food service intervention targeted the school shops and the lunch boxes sent by the family. The family program consisted of meetings, health fairs as well as information packets were sent home. Also, parents participate in the focus group	Dietary, PA, and SB habits (questionnaire)	There was no difference in PA and screen time habits and no changes in BMI between groups -post-intervention. Students in the IG were 40% more likely to play at recess during post-test compared with control students, but changes in the levels of play at home, an after-school sports activity, and screen time habits, were not observed	PA: Nothing, SB: Nothing, F/S: Nothing
Fairclough et al, 2013	To investigate the effectiveness of the CHANGE project, to promote healthy weight using an educational focus on PA and healthy eating	318; 10-11 years old; Wigan Borough, England	20 weeks intervention. “CHANGE!” program was based on formative work conducted with teachers, children, and parents. Teachers from the intervention schools received 4 hours of training. 20 weekly lesson plans, worksheets, homework tasks, lesson resources, and a CD-ROM. 60 minutes duration and provided an opportunity for children to discuss, explore, and understand the meaning and practicalities of PA and nutrition. The program provides homework tasks supplemented the classroom work and targeted family involvement in food and PA-related tasks. They provide formative work to parents	Intensity of PA (Accelerometer used 7 days)	At follow-up there was a significant intervention effect for LIPA (β=25.97 (95% CI = 8.04, 43.89) min, P=.01). The intervention was most effective for overweight/obese participants, girls, and participants with higher family socioeconomic status	PA: Positive, SB: Nothing, F/S: Nothing
Muros et al, 2013	To determine the effect of nutritional education given to children and their parents combined with sessions of vigorous extracurricular, PA on the improvement of health-related parameters	IG 25 (15 M/10F); CG 29 (10 M/19F), 10-11 years old; Southern Spain	7 weeks intervention. The intervention program included. Thirteen 60-min sessions of vigorous extracurricular PA held twice a week. Nutritional education sessions lasting approximately 2h each were provided to both students and their parents with parents completing 4 and the student completing 2. Parents participate in the nutritional education sessions	Aerobic Capacity (Shuttle Run). Healthy Habits Survey (to measure physical, psychological, and nutritional status)	For CG there were no significant differences between pre-test and post-test, however, the IG showed significant (P < .01) improvement after the program in the maximum oxygen uptake and dietary intake profile. In the healthy Habits questionnaire, the IG showed increased levels of PA relative to the pre-test	PA: Positive, SB: Nothing, F/S: Nothing
Wright et al, 2013	To evaluate the impact of a nurse-directed, coordinated, culturally sensitive, school-based, family-centered lifestyle program on PA and BMI.	251 (150F, 101M); 8 – 12 years; Los Angeles, USA.	6-week duration. The Kids N Fitness (KNF©) intervention provided 45 min of structured PA peer week for children and 45 min nutrition education class for parents and children. School-wide wellness activities, including health and counselling services, staff professional development in health promotion, parental education newsletters, and wellness policies for the provision of healthy foods at the school. Parents participate in the education class and fulfilled the demographic questionnaire	Activity behaviors (CATCH SPAN questionnaire, utilizing 5 items – daily PA, participation in team sports, attends PE class, TV computer/video game use)	Significant results for students in the intervention included for boys decreases in TV viewing; and girls increases in daily PA, physical education class attendance, from baseline to the 12-month follow-up	PA: Positive, SB: Positive, F/S: Nothing
De Coen et al, 2012	To evaluate the effects of a school-based, 2-year, multi-component intervention on BMI, eating, and PA behaviour in communities of high and low socio-economic status (SES)	IG (396 parents and 670 children’s); CG (298 parents and 442 children’s) 3-6 years; Flanders, Belgium	2 years intervention. The healthy eating and PA intervention was based on the socio-ecological model in health promotion programs with the child as the center of focus situated within several layers (family, friends, pre-primary or primary schools, community stakeholders, local policy, and media). The intervention materials for the parents were newly developed for the project. The parents received a poster visualizing the target messages and containing short tips regarding parenting practices and styles to encourage children to stick to the healthy eating and PA targets	PA (questionnaire fellfield by parents, to measure the participation of the children in sports club or school sports activities, h/week, scream time spent)	There were no significant intervention effects founded for eating behaviour, PA, or screen-time; there were no significant interaction effects of age and gender of the children on the outcome variables	PA: Nothing
						SB: Nothing
						F/S: Nothing
Kargarfard et al, 2012	To examine the effectiveness of parental support and involvement intervention for the improvement of health-related fitness of high school girls through an after-school PA program	IG 206 children and 204 mothers (aged 15.8±1,0 years old); CG 60 children and 60 Mothers (aged 15.9±1,3 years old); 7 provinces in Iran	12 weeks intervention. This after-school PA program on health-related fitness of mother/daughter pairs (CASPIAN) was based on 24 sessions, 90 min (including 60-70 min of fitness orientated activities), for 2 afternoons a week. Fitness activities consisted of a 10-min warm-up, 30 min of aerobic activity and stretching exercises, 20 min free group playing and 10 min of cool down. Mothers participated in the full intervention group	Health-related fitness (1-mile Walk Test, Sit-and-Reach Test, Push-Ups Test and Sit-Ups Test)	There were significantly improved, cardiorespiratory fitness, flexibility and muscle strength, and endurance in children in both groups, and mothers. There were significant improvements in the physiological measures of the children in the mother/daughter group	PA: Nothing
						SB: Nothing
						F/S: Positive
Dzewaltowski et al, 2009	To assess the effectiveness of a multilevel intervention model designed to develop the skills and efficacy of adult leaders and youth to build middle school environments that promote vegetable consumption and PA.	IG 767 (54.18% F) 12.36±40 years old; CG 815 (53.6% F), 12.40±43, USA.	3 years. The HYP intervention targeted increased fruit and vegetable consumption and PA through building the environmental change skills and efficacy of adults and youth. The intervention model was designed to influence proxy efficacy by building youth’s confidence that they could influence others, teachers and parents, to assist them in building healthy places	PA levels by PDPAR.	After the intervention, HYP schools did significantly change in PA compared to control schools. Proxy efficacy to influence school PA environments mediated the program effects. Building the skills and efficacy of adults and youth to lead school environmental change may be an effective method to promote youth PA.	PA: Positive SB: Nothing F/S: Nothing
Jan et al, 2009	To assess the impact of Shape it up program, a health education intervention to promote knowledge and positive, Attitudes toward exercise and healthy eating among elementary, School children	6421 (49.9% Males); Don’t say the age, only children’s form the 2° to 5° grade; New Jersey, USA.	2 weeks intervention. Shape it up program was a 60-minute interactive workshop of healthy eating and exercise. Students who attended also received a Shape it up booklet to take home and share with family members. Posters that presented key intervention messages for display in classrooms, school hallways, and cafeterias. Field days, after the school intervention, of active games based, along with consumption of healthful foods and beverages. They create a website for parents or guardians could visit for further information about the program and healthy lifestyles	Attitudes about healthy eating and exercise (questionnaires)	Children reported higher levels of knowledge and positive attitudes about healthy eating and exercise compared with the baseline survey results. Appears to have had a positive impact on children’s knowledge and attitudes toward exercise and healthy eating	PA: Nothing
						SB: Nothing
						F/S: Positive
Engels et al, 2005	To examine the effectiveness of a unique inner-city middle-school–based, extracurricular, After-school program, To foster both, Healthful diets and exercise patterns	56 children’s (18 M/38F; age 11.1 ±1.3 year); 25 parents (25F; age 40.6±7.7 years); urban African American	12-weeks intervention. The extracurricular after-school program included 4 days each week, 60 to 75 minutes per session including the provision of supervised dance, sports games, fitness activities, step pedometers, established 5 A Day for better health program nutrition learning activities. The parents participated in the after-school program and helped to fulfil the frequency of fruit and vegetable intake	Fitness run/walk test (1.65 miles on indoor track)	No improvement was seen in children’s fitness. Parents/guardians showed improvements in endurance walk/run time (*P* < .05). Findings indicate that children tended to gain more diet-related benefits while parents/guardians tended to derive more fitness-related benefits	PA: Nothing
						SB: Nothing
						F/S: Positive

Abbreviation: CG, control group; CPAIQ, children PA Item questionnaire; EPAQ, eating and PA questionnaire; GOAL, Guys/Girls Opt for activities for life; healthy youth Places, HYP; HEELP, exercise, eating, and lifestyle; HR, heart rate; HRQol, health-related quality of life; IG, intervention group; LIPA, light physical activity; METs, metabolic equivalent; MVPA, moderate to vigorous physical activity; PA, physical activity; SB, sedentary behaviour; F/S, fitness/skills; PDPAR-24, previous Day PA Recall; PedsQL, Pediatric quality of life Inventory; VPA, vigorous physical activity

Most studies performed a multicomponent approach (n=20), intervening in nutrition education, physical activity, psychological variables, and/or re-education for healthier lifestyle behaviours. In the other 2 studies^31,33^ the intervention programs focused on promoting physical activity. Also, different forms of family involvement were employed. Half of the studies (n=11)^21,23-25,28-30,34,37-39^ involved the family through sessions/workshops, in which tools and knowledge about healthy lifestyles were provided. In 8 studies,^22,27,32,33,35,40-42^ parental involvement focused on carrying out/completing assessment instruments (ie, questionnaires) and/or on participating in some intervention processes (eg self-monitoring; family homework). The other 3 studies^26,31,36^ contemplated a direct parental involvement in physical activity, where parents performed physical activity with their children.

### Main Results

The main results of each study are presented in Table 2. The outcomes considered in this systematic review were organized into 3 components: physical activity, sedentary behaviour, and fitness/skills. Most studies (n=19) showed a positive intervention effect in at least 1 of the outcomes of interest (physical activity, sedentary behaviour, fitness/skills), with only 3 studies^22,23,28^ not reporting post-intervention improvements. Ten studies reported improvements in physical activity behaviour.^25,27,29,30,32,36,37,39,41,42^ From those, 4 studies additionally revealed improvements in sedentary behaviour^27,36,39,41^ and 1 study presented further improvements in sedentary behaviour and fitness/skills.^42^ Other 8 studies showed positive effects of the intervention only on fitness/skills.^21,24,31,33-35,38,40^ Lastly, only 1 study reported improvements solely in sedentary behaviour.^26^

Some characteristics of the studies appeared to influence the main results. For example, all the 3 studies that included direct parental involvement in physical activity^26,31,36^ showed an improvement in 1 or more outcomes. Also, all studies encompassing more than 1 educational level showed a positive effect of the intervention on the outcomes of interest. On the other hand, all interventions targeting children aged 6 years or less^22,23^ showed no significant impact.

## Discussion

This study systematically reviewed the effects of school-based interventions involving the family in promoting physical activity, reducing sedentary behaviour, and improving fitness/skills among children and adolescents. Of the 22 included studies, 19 showed a positive effect in at least 1 of the main outcomes of interest. Most interventions employed a multicomponent approach, integrating physical activity promotion, nutrition education, and general education for healthier lifestyle behaviours. Shorter intervention durations and greater family involvement appeared to be more effective.

Although there is some evidence on the effectiveness of interventions to promote physical activity in the school setting,^25,39,41^ it is necessary to understand further the factors that support the change and maintenance of healthy behaviours, such as physical activity, in the medium- and long term. In the reviewed studies, we found an association of other areas of intervention with the main purpose of promoting physical activity to enhance the acquisition of different behaviours and skills. Those areas mostly integrate nutrition and the motivational component.^23,32,38,40^ Multicomponent and multidisciplinary interventions that involve physical activity and combine nutritional education to promote behaviour changes and redefine parenting strategies are considered the main potential components for creating an intervention program.^43,44^ Psychological variables such as knowledge, motivation, mental well-being, and psychosocial aspects are also sometimes associated with this type of intervention.^45^ However, the results are still very incipient, so further studies are needed to understand better the effectiveness and mechanisms of multidisciplinary interventions for physical activity promotion in school contexts involving the family. This is also relevant when sedentary behaviour and fitness/skills outcomes are considered.

Discussing the best way to involve the family in learning and acquiring skills in the school context is not a recent issue.^46-49^ There is some evidence that the lack of support from family reduces the likelihood that behaviours and skills acquired in the context of regular physical activity will persist over time.^50,51^ The importance of family involvement in interventions in health and maintenance of more active and healthier lifestyles is widely recognized. Even so, interventions centred on the family have different forms of structuring. While some choose to focus on parents as the sole agents of change, strengthening parents’ leadership skills,^52,53^ others promote parental support in changing the child’s behaviour, taking responsibility for creating a supportive environment, and empowering children to make informed choices.^53,54^ Ideally, from a public health perspective, interventions designed based on simultaneous participation and pursuit of goals by the family and children, making commitments to change behaviour together, are the most sustainable.^55,56^ However, only 3 studies in this review^26,31,36^ achieve more interactive and direct participation by the family concerning inclusion in the practice of physical activity, with consistent results. Factors such as labour issues, lack of time, or inflexibility of schedules, among others, may justify a certain inability to generate contexts and intervention programs that do not clash with the existing daily routine.^50,57,58^

In the revised literature, the inclusion of the family in a perspective of control and evaluation of the process, acquisition of skills and knowledge that regulate and encourage physical activity practice occurs mainly through sessions/workshops to transmit knowledge about healthy lifestyles and filling in questionnaires or reports. It appears to be the most used way to promote and generate interventions to increase physical activity levels, both in children and families. Although the literature indicates the importance of parental involvement and the creation of specific conditions in the family context as critical aspects for the success of the interventions,^59,60^ it has been common to find a high heterogeneity among existing studies, both in methodological terms in intervention strategies. These factors highly limit the results’ applicability and consequently do not provide sufficient evidence to obtain political relevance to support or improve this type of program.^61,62^

Another important variable to consider in the study’s design and structure is the age and/or school years for which the programs are designed. It seems evident that intervention programs are mainly focused on younger children. The nature of interactions between parents and their children’s school becomes more formal and less frequent after the first few years of schooling.^63^ It is also important to realize that the younger the children’s ages, the greater the possibility that the programs will depend on families' involvement in aspects unrelated to the exercise itself, but related to other components such as evaluation processes, filling out questionnaires, and others. On the other hand, as children are getting older, there are many factors to consider, such as more difficulties in changing behaviours and lifestyles more significant number of affordances and behaviours different from those that the programs intended to promote. In addition, when compared to adolescents, younger children may be more flexible in their ability to change behaviours since it is at this stage that they begin to develop self-regulation skills for a healthy life.^64^ How Parent and family support tends to peak at age,^12,65^ and there is a greater tendency for teenagers to become independent in their leisure time and follow influences from their peers.^66^ Considering this, and since the results showed that children under 6 years old did not show significant effects in the interventions, it is believed that the ideal interval to develop this type of intervention program would be between the age of 6 years to adolescence.

Regarding the duration of interventions, most studies reviewed have fewer than 3 months (n=10) or are longer than 12 months (n=9). Concerning the motivation and adherence factors of the program’s interventions, short-term interventions have greater effectiveness in physical activity, physical fitness, and/or skills related to healthy behaviours and lifestyles than longer interventions duration. In sedentary behaviour, a similar number of studies showed positive results. The lowest rate of studies analysed lasted 3 to 6 months with manifestly reduced effects on study outcomes. These results can be important indicators of the difficulty of maintaining the positive impact of interventions for more extended periods. Strategies to strengthen the, especially in long-term interventions, are essential factors.

### Limitations

The findings of the current systematic review should be interpreted considering some limitations. Although study quality was assessed, studies were not weighted or ranked, nor were any removed from the review. Therefore, studies with poorer quality were given no less importance than findings from studies with greater quality. Furthermore, the studies included presented a variety of instruments for assessing the outcomes. In addition, 1 study did not clarify the family’s role in the intervention programs.^24^ It is important to note that only published articles were included in this systematic review. In this way, the publication bias may also be a limitation since there may have been unpublished studies not included due to not having achieved significant results. Finally, there was high heterogeneity among included studies, both in methodological terms and intervention strategies. All these factors lead to difficulties and caution in the comparisons presented. The impact of context constraints on the design of the intervention (eg, locals’ educational policies) and the multidisciplinary approach put a severe challenge to the precision of future intervention implementation (ie, repeatability and reproducibility).

## Conclusion

School-based interventions involving the family seem to effectively promote physical activity behaviour, reduce sedentary behaviour, and improve fitness/skills. Successful intervention programs should include a multicomponent and multidisciplinary approach, integrating physical activity promotion, nutrition education, and general education for a healthier lifestyle. Family support increases the likelihood that behaviours and skills acquired in regular physical activity will persist over time. However, few interventions achieve more interactive and direct participation by the family concerning inclusion in physical activity practice. Future interventions should overcome this limitation by integrating participation and pursuit of goals by the family and children simultaneously, making commitments to change behaviour together. Due to the high heterogeneity among studies, both in methodological terms and intervention strategies, more studies are needed to understand better which factors impact the success of the physical activity, sedentary behaviour, and fitness/skills programs.

**So What?**

Implications for Health promotion Practitioners and Research

**What is already known on this topic?**

There is still no systematized evidence that reports the influence of School-based interventions involving the family on children and adolescents’ physical activity levels.

**What does this article add?**

This systematic review provides new information on how physical activity interventions centred on the family at the school can increase physical activity and reduce sedentary behaviour. We considered the level of physical activity, sedentary behaviour, and fitness/skills at the school context as the pillars of this research. Given this three-dimensional scenario, it was possible to identify standardised dynamics concerning how successful interventions should be structured.

**What are the implications for health promotion practise or research?**

School-based interventions involving the family seem to effectively promote physical activity behaviour, reduce sedentary behaviour, and improve fitness/skills. This approach requires a multicomponent and multidisciplinary method, integrating physical activity, nutrition education, and general education for a healthier lifestyle. Also, interventions designed based on simultaneous participation and pursuit of goals by the family and children, making commitments to change behaviour together, are the most sustainable.
